# Deep Diving Into the Cardiovascular Health Paradox: A Journey Towards Personalized Prevention

**DOI:** 10.3389/phrs.2024.1606879

**Published:** 2024-07-17

**Authors:** Bamba Gaye, Nabila Bouatia Naji, Mario Sims, Yendelela Cuffee, Oluwabunmi Ogungbe, Erin D. Michos, Camille Lassale, Pierre Sabouret, Xavier Jouven

**Affiliations:** ^1^ Alliance for Medical Research in Africa (AMedRA), Department of Medical Physiology, Cheikh Anta Diop University, Dakar, Senegal; ^2^ Université Paris Cité, PARCC, INSERM, Paris, France; ^3^ Department of Medicine, University of Mississippi Medical Center, Jackson, MS, United States; ^4^ Alliance for Medical Research in Africa (AMedRA), Epidemiology Program, University of Delaware, Newark, DE, United States; ^5^ Johns Hopkins University School of Nursing, Baltimore, MD, United States; ^6^ Division of Cardiology, Johns Hopkins University School of Medicine, Baltimore, MD, United States; ^7^ Alliance for Medical Research in Africa (AMedRA), Barcelona Institute for Public Health (ISGlobal), Barcelona, Spain; ^8^ Universitat Pompeu Fabra, Barcelona, Spain; ^9^ CIBER of Physiopathology of Obesity and Nutrition (CIBEROBN), Institute of Health Carlos III, Madrid, Spain; ^10^ Heart Institute, Pitié Salpétrière Hospital, Sorbonne University, Paris, France; ^11^ National College of French Cardiologists, Paris, France; ^12^ Assistance Publique-Hôpitaux de Paris, Georges Pompidou European Hospital, Cardiology Department, Paris, France

**Keywords:** cardiovascular health, precision prevention, personalized approach, emerging risk factors, CVH paradox

## Abstract

**Objectives:**

The Life’s Simple 7 score (LS7) promotes cardiovascular health (CVH). Despite this, some with optimal LS7 develop cardiovascular disease (CVD), while others with poor CVH do not, termed the “CVH paradox.” This paper explores pathways explaining this paradox.

**Methods:**

We examined methodological aspects: 1) misclassification bias in self-reported lifestyle factors (smoking, physical activity, diet); 2) cumulative exposure to risk factors over a lifetime, impacting the CVH paradox. Punctual risk factor assessments are suboptimal for predicting outcomes. We proposed personalized prevention using “novel” elements to refine CVH assessment: 1) subclinical vascular disease markers, 2) metabolic biomarkers in blood and urine, 3) emerging risk factors, 4) polygenic risk scores (PRS), 5) epigenetics, and 6) the exposome.

**Results:**

Addressing the CVH paradox requires a multifaceted approach, reducing misclassification bias, considering cumulative risk exposure, and incorporating novel personalized prevention elements.

**Conclusion:**

A holistic, individualized approach to CVH assessment and CVD prevention can better reduce cardiovascular outcomes and improve population health. Collaboration among researchers, healthcare providers, policymakers, and communities is essential for effective implementation and realization of these strategies.

## Introduction

Over the last 4 decades, there have been major advances in the prevention of cardiovascular disease (CVD) that have helped reduce CVD incidence and related mortality [1]. However, CVD remains the leading cause of mortality worldwide, and is projected to increase from 17.8 million in 2017 to 23.3 million in 2030 [2–4]. Therefore, CVD prevention and promotion of cardiovascular health (CVH) remain a public health priority.

The American Heart Association (AHA) proposed the Life’s Simple 7^®^ score to assess overall CVH. It is comprised of 7 modifiable risk factors that are classified as poor, moderate or ideal; generating a score ranging from 0 to 14, with 14 representing an optimal CVH, i.e., no smoking, regular physical activity, healthy diet, and normal body mass index (BMI), blood pressure (BP), cholesterol, and glucose values [5, 6]. Accordingly, multiple studies have reported an association between greater CVH scores and lower risk of CVD and various chronic disease outcomes [6–10]. Furthermore, improvement from poor to intermediate/ideal CVH have been shown to be associated with a lower risk of CVD and mortality [11].

Despite the reported inverse associations between CVH score and CVD risk, some individuals with optimal CVH may develop CVD. In other words, they have “residual risk.” Conversely, some individuals with poor CVH may never develop CVD. We term the aforementioned phenomenon the “CVH paradox” suggesting that currently established risk factors cannot fully explain CVD risk. This CVH paradox, by analogy, is closely related to the well-established concept of residual risk, which refers to the remaining risk of CVD after patients have been fully treated for their disease. Recent findings show CVD prevalence in persons classified as having ideal cardiovascular health vary between 11% and 24% [12]. Inversely, around 40% of persons with poor cardiovascular health never develop CVD [12]. Understanding the risk discrepancies between those in ideal cardiovascular health who do develop CVD and those who do not (and inversely, between those in poor health who never develop CVD and those who do) is key to defining individual risk for CVD.

This would shift the narrative towards a personalized, preventive approach, whereby the aim could then become targeting individuals for risk prevention before they are even considered at risk for CVD.

## Methods

This review aimed to explore the pathways contributing to the cardiovascular health (CVH) paradox and propose personalized prevention strategies to improve cardiovascular outcomes. To achieve this, we conducted a comprehensive literature review following these steps:

### Literature Search

We performed a systematic search of PubMed databases to identify relevant studies published between 2010 and 2023. The search terms included combinations of keywords such as “cardiovascular health,” “Life’s Simple 7,” “CVH paradox,” “misclassification bias,” “cumulative exposure,” “subclinical vascular disease,” “metabolic biomarkers,” “polygenic risk scores,” “epigenetics,” and “exposome.”

### Inclusion and Exclusion Criteria

We included peer-reviewed articles, review papers, and meta-analyses that:

1. Discussed related concepts.
2. Explored methodological aspects such as misclassification bias in self-reported lifestyle factors.
3. Investigated the impact of cumulative exposure to risk factors on cardiovascular outcomes.
4. Examined novel elements of personalized prevention, including subclinical markers, metabolic biomarkers, emerging risk factors, polygenic risk scores, epigenetics, and the exposome.

Studies were excluded if they:

1. Were not published in English.
2. Focused solely on single-point risk factor assessments without considering cumulative exposure.
3. Did not provide substantial data or insights related to the CVH paradox or personalized prevention strategies.

### Data Extraction and Synthesis

We extracted relevant data from the included studies, focusing on:

1. Definitions and descriptions of the CVH paradox.
2. Evidence and examples of misclassification bias in self-reported lifestyle factors.
3. Findings on the impact of cumulative exposure to cardiovascular risk factors.
4. Identification and evaluation of novel personalized prevention elements.

We synthesized the extracted data to identify common themes, gaps in the literature, and potential pathways contributing to the CVH paradox. The findings were organized into thematic sections, including misclassification bias, cumulative exposure, and personalized prevention elements, to provide a comprehensive overview and propose future research directions.

### Review and Analysis

The synthesized data were reviewed and analyzed to highlight the implications for clinical practice and public health. We proposed a conceptual framework integrating traditional risk factors with novel personalized prevention elements to address the CVH paradox and improve cardiovascular outcomes. The review findings were discussed in the context of current prevention strategies, with recommendations for future research and practical applications.

## Results

### The Cardiovascular Health Paradox

Although associations between established risk factors and CVD morbidity and mortality have been reported extensively, understanding how other factors may affect a person’s CVH can help provide a more individualized approach to disease prevention and treatment. However, providing advice that is tailored to each individual based on their environmental, social, and genetic background may be challenging, especially if those individuals are considered to be in an overall healthy state. Identifying environmental exposures and novel disease mechanisms, such as epigenetic modifications, can help discover a set of therapeutic targets useful for directed screening and ultimately achieving better treatment for every individual.

The aim of this study is to provide a review of the determinants of individual cardiovascular risk that could explain the presently described cardiovascular health paradox.

### Misclassification Bias

A possible explanation for the residual risk is the misclassification bias for self-reported lifestyle factors (i.e., smoking, physical activity, and diet). People tend to over-report healthy habits, and therefore, present with a high CVH score [13, 14]. However, some of these individuals are in fact likely to have poor CVH and at risk of CVD, and some individuals sharing the same CVH score may be differently impacted in regard of likelihood incidence of cardiovascular events. Moreover, non-modifiable risk factors such as age, sex and family history are not comprehensively incorporated in the CVH score but play an important role in CVD risk. Age being the most important predictor of CVD risk, the relative added predictive value of the 7 CVH modifiable factors may be hindered at older ages. Although these explanations can partially justify the discrepancy between CVH score and CVD risk, an array of other biological explanations should be explored [15].

### Cumulative Exposure to Risk Factors

Another critical trope that should be addressed within the paradox is the notion of cumulative time-varying exposure to risk factors, and its impact on cardiovascular health. Fixed risk factors can not explain the health paradox. If fixed risk factors are not enough to explain the health paradox observed in cardiovascular prevention strategies, then surely other dimensions, particularly those that are not binary, need to be studied further.

One of these dimensions is cumulative exposure. To this day, our knowledge of cardiovascular risk factors worldwide (smoking, physical activity, patient and family history, dyslipidemia, elevated blood pressure, diabetes, obesity, sex and age), fails to address the impact of cumulative exposure in cardiovascular risk factors. For example, cholesterol is a known risk factor for cardiovascular events. Recent studies are increasingly showing [16] that independently of midlife LDL-C levels, the cumulative presence of LDL-C and during young adulthood is a risk factor in and of itself [17]. This is especially true in patients with familial hypercholesterolemia [17], where the cumulative factor of LDL-C presence has been proven to be independently strongly associated with the occurrence of major cardiovascular events. Therefore, Ference et al. suggested that monitoring of atherosclerotic plaque burden progression may be relevant to assess clinical benefit of primordial prevention, and provide individuals evidence of benefits from maintaining optimal lipid levels [18].

Similarly, cumulative time-varying exposure to elevated blood pressure has also been shown to increase the risk of CVD regardless of fixed measurements [19]. This again suggests that our classification of cardiovascular risk considers the lifetime-evolution of blood pressure for each individual so as to better anticipate their risk of developing CVD [20].

Therefore, documenting a reduced rate of atherosclerotic plaque burden progression, rather than a reduced incidence of clinical cardiovascular events, may be a more intuitive and accurate metric for assessing the clinical benefit of primordial and primary prevention. Using this metric, nearly every person can assess how much he or she is benefiting from maintaining optimal lipid levels. C-reactive protein has also become a topic of interest as cumulative time exposure: new studies are proving that high sensitivity CRP is associated in a dose-response pattern to increased cardiovascular risk and myocardial infarction, depending on the number of years exposed to elevated levels of CRP-mediated inflammation [21].

Another promising lead to explain the health paradox in relation to cumulative exposure, is to evaluate the accumulation of multiple risk factors over time, rather than one element only. This is particularly true for risk factors that are composite. Cumulative social risk, for instance, is in part a consequence of environmental pollutants, cumulative exposure to bad air quality and PM_2.5_ pollutants in addition to socio-demographic risk factors. These are now being shown to increase overall social risk for CVD and mortality [22]. Increasing numbers of social risk factors is proportionally linked to the unlikeliness of ideal cardiovascular health [23].

Another example is impaired fasting glucose, a type of pre-diabetes, impaired fasting glucose is largely mediated by the combined presence of dyslipidemia and hypertension; and this combined presence has manifested itself to be independently associated with CVD risk [24]. The fact that two independent risk factors can create a third is a pattern that deserves to be studied further so that guidelines for prevention can adapt to the notion of cumulative exposure and its associated cumulative risk.

Furthermore, this pattern has exhibited molecular sense. Epigenetic modules could indeed be implicated [25] as a response to the cumulative exposure to traditional cardiovascular health determinants. This would help explain the physiopathology behind the theory of cumulative exposure.

Overall, the impact of risk accumulation over time is a notion that already has been used in practice before. Smoking, for instance, is evaluated in number of pack-years smoked rather than in binary features. The case of smoking has proved many times that cessation is not enough to annihilate the added cardiovascular risk completely [26]. Extrapolating to the theory of cumulative exposure, this suggests it could be promising to evaluate not only the impact of accumulation on risk levels, but also on the effectiveness of cessation. Furthermore, the study of cumulative risk is promising in terms of discovering new risk factors outside of traditional health determinants. Some factors may only have an impact when accumulated over time. This is already the case with reproductive risk factors, such as postmenopausal status in women, for example, which have been shown to decrease the likeliness to reach ideal cardiovascular health [27].

Overall, studying cumulative exposure is promising in the impact it could have on the discovery of factors that have so far been studied solely in a fixed, and not cumulative, manner in regards to cardiovascular health, hence furthering our understanding of the cardiovascular paradox.

### The Promise of Personalized Prevention

#### Methodological Aspects

CVD prevention and treatment strategies are targeted at individuals classified as high or moderate risk, based on existing algorithms such as the Framingham risk score, Pooled Cohort Equations (PCE), or SCORE in Europe as recommended by the European Society of Cardiology guidelines [28], all of which include very well-established and standardized CVD risk prediction scores. This uniform approach to care at a broad group level is challenged by a National Institutes of Health statement that precision medicine “is an emerging approach for disease treatment and prevention that takes into account individual variability in environment, lifestyle, and genes for each person” [29]. Precision medicine has already been applied to early disease detection and treatment for cancer, asthma, chronic obstructive pulmonary disease (COPD), and other disease outcomes [30, 31]; but there has been increased interest in applying these same principles to CVD prevention [32]. A personalized life course approach provides individualized preventive advice and treatment according to an individual’s personal characteristics, lifestyle, and background. CVD may be affected more by one’s environment than one’s genetics, hence emphasizing the need to investigate the ways and conditions under which different biological pathways interact [15]. Understanding the multidisciplinary dynamics behind these pathways and assessing their predictive value can help explain the CVH paradox.

**FIGURE 1:**
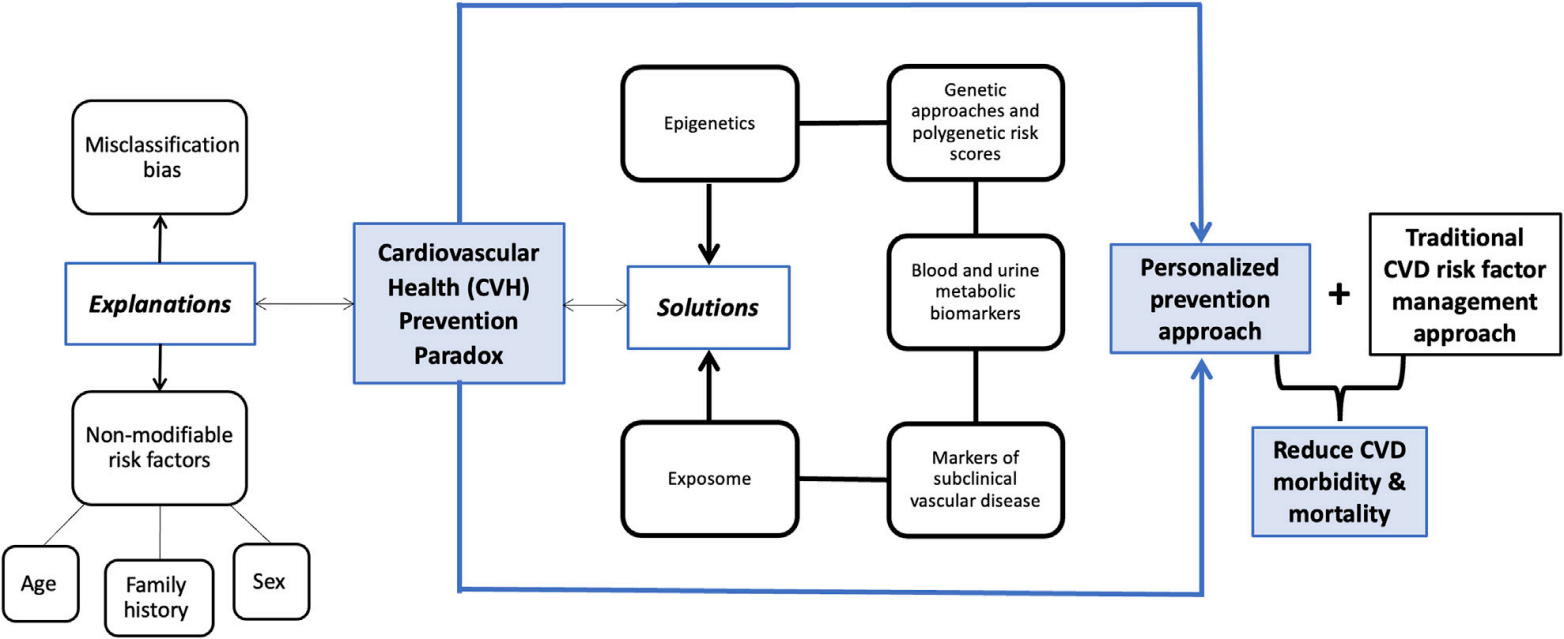
Conceptual framework demonstrating different explanations and solutions for the cardiovascular health (CVH) paradox (Deep diving into the cardiovascular health paradox: a journey towards personalized prevention. Senegal, 2024).

Reaching an optimal CVH score is associated with healthy living and has shown to successfully reduce the risk of CVD over time. However, the population-wide prevention method leaves out a proportion of individuals with unexplained CVD. Shifting towards an individualized multidisciplinary approach can help reduce the “residual risk” and, in turn, allow for a more inclusive management of cardiovascular care. The complexity of the CVH paradox leaves room for several explanations and therefore, several solutions that should be implemented in CVD prevention. We will highlight possible explanations and solutions to the CVH paradox, including markers of subclinical vascular disease, blood and urine metabolic biomarkers, polygenic risk scores, epigenetics, and exposmome, and we will emphasize the positive impact of adopting a personalized prevention approach (Figure 1).

#### Markers of Subclinical Vascular Disease

Subclinical markers of vascular disease may be independent predictors of CVD events. Firstly, the calcium coronary score is acknowledged to reflect the lifetime effects of both measurable and non-measurable risks factors, directly through the coronary artery calcium, and may guide aspirin or statin prescriptions [33–35]. Moreover, it is used to personalize management of comorbidities such as diabetes. Nonetheless, it requires the use of irradiation through CT scan, and is not yet a routine international recommendation.

Indices of arteriosclerosis/arterial stiffening include reduced brachial reactivity, abnormal pulse wave velocity, carotid distensibility, Young’s elastic modulus, common carotid artery calcification, or retinal vessel diameter as a microvascular biomarker [8]. Similarly, indices of atherosclerosis include carotid plaques, coronary artery calcium, reduced ankle brachial index [8].

Moreover, flow-mediated dilation is a gold standard method for measuring endothelial function [36, 37]. Using this non-invasive method can help prevent the development of atherosclerotic disease among people who are otherwise not considered at risk for developing CVD [36, 37].

Beyond markers of vascular health, markers of chronic kidney disease (albuminuria and eGFR), which is a strong risk factor for CVD, may be used to quantitatively enhance CVD risk prediction [38]. Studies found that the majority of individuals with subclinical atherosclerosis are not classified as having inadequate CVH or as high-risk with the Framingham Risk Score [39]. Subclinical markers of vascular disease provide measures of vascular aging and are present to various degrees depending on disease chronology amongst other factors, prior to having symptoms of vascular disease. Therefore, they can serve as useful tools for early detection of CVD in people who would otherwise be classified as moderate or low risk, but would benefit from targeted pharmacological or lifestyle intervention.

#### Blood and Urine Metabolic Biomarkers

Circulating inflammatory and hemostatic biomarkers such as high sensitivity C-reactive protein (CRP), interleukin-6 (IL-6) and fibrinogen are the “usual suspects” and have been consistently associated with risk of CVD and stroke [40–43]. Their added predictive value has been shown to be moderate but potentially useful in the case of CRP and fibrinogen [44]. Beyond increments in prediction, few studies have explored the potential mediating role of such circulating biomarkers in the association between CVH and CVD. Using data from the Framingham Offspring Study, a study concluded that the lower risk of CVD associated with optimal CVH is partly mediated by lower inflammatory (CRP and IL-6) and hemostatic (fibrinogen) blood biomarkers [9]. Apart from inflammatory biomarkers, other promising biomarkers can be grouped as thrombotic (e.g., homocysteine, lipoprotein-associated phospholipase A2), glucose-related markers (e.g., HbA1c), lipid-related markers (e.g., apolipoproteins) and organ-specific markers (e.g., kidney, cardiac). More complex and not routinely assessed markers are being explored, such as markers of HDL functionality, metabolomics or proteomics signature of underlying cardiometabolic disorders [45]. For example, Hoogeveen and colleagues found a proteome-based model to be significantly more effective in predicting CVD than clinical risk factors [46]. The study looked at a panel of 50 proteins, most of which are related to immune response, and matched data from Framingham risk score in order to ensure the accuracy and reliability of the CVD predictions [47]. Meta-analyses and systematic reviews, however, suggest that the vast majority of other circulating and urinary biomarkers have no or limited proven ability to improve risk classification [28].

#### Genetic Approaches and Polygenic Risk Scores

The interaction between genetics, environment, and etiologic heterogeneity, has allowed for a new and more definitive understanding of CVD prevention among high-risk individuals [47]. It also provides an explanation on how genetics can cause certain diseases through environmental interactions, which can help determine the most effective targeted prevention strategies over the course of disease evolution [47]. In the absence of known risk factors, individuals can be at high risk of CVD due to genetic makeup. Family history of premature CVD data is inexpensively and easily collected and has value as a risk modifier in people for whom estimated CVD risk borders on a decisional threshold [28]. Robert Runnels Williams and colleagues explored how gene therapy can influence the diagnosis of familial forms of atherosclerosis, hypertension, and thrombosis and how practical application of targeted gene strategies can prevent CVD [48]. An analysis of the UK Biobank study concluded that both a polygenic risk score and health behaviors contributed to CVD risk and that genetic risk scores were associated with the risk of CVD regardless of health behavior and environment [49]. This finding further explains the CVH paradox and promotes the concept of precision medicine in the field of prevention, particularly primordial prevention. Assessing polygenic CVD risk can be done once in a lifetime and may help identify risk early on in younger patients with otherwise optimal CVH [48–51]. It has been shown that such identification can help mitigate the excess risk associated with genetic disorders by actioning appropriate pharmacological and lifestyle modification strategies [52]. However, despite the ever increasing affordability and availability of genome wide assessment at the individual level, existing data show only modest improvement of prediction by including polygenic risk scores in CVD risk algorithms [53]. This, and the lack of agreement regarding the choice and calculation of genetic risk scores make their use in routine care not recommended in current clinical guidelines.

#### Epigenetics

Epigenetics interconnects an individual’s genetic makeup and external influences to explain the progression of CVD. Two main epigenetic mechanisms include DNA methylation and non-coding RNAs. Regarding DNA methylation, on the one hand, studies have identified a number of DNA methylation markers related to CVD and its risk factors, in particular obesity [54–56]. On the other hand, epigenome wide association studies have identified differential methylation at specific CpG loci associated with smoking, physical activity, and diet [57, 58]. Further research is needed to clarify whether they are mediators of the methylation-CVD association and the importance in determining the independent association of epigenetics and CVD risk, hence furthering our understanding of life course cardiovascular prevention.

Mutations of certain non-coding RNA (ncRNAs) such as micro RNA (miRNA) are involved in CVD development [59]. These molecules are now considered possible targets for intervention [60]. According to Zampetaki and colleagues, three miRNAs were considered as targets for detecting myocardial infarctions (MI) [61]. Specifically,miR-126 was found to have a significant association with incidence of MI [62]. In this context, the discovery of an entirely new method of recognition and regulation by ncRNAs, and their validation as markers and modulators of pathological conditions, provides hope for innovative disease diagnosis and therapy [63]. Experimental data suggests that circulating ncRNAs could be relevant biomarkers to detect early stages of atherosclerosis, but epidemiological evidence remains scarce [58]. The recent promising technical advance in miRNA in cluster analysis (Maximum Weighted Merger Method [62], microarray technology [63], to name a few) complete the picture of the potential role of genomics on CVD risk at the population level [64].

#### Exposome

The exposome is defined as “the totality of human environmental exposures from conception onwards” [65]. It includes a variety of exposures such as education, residential environment (e.g., urban vs. rural, deprivation), built environment, occupational environment (e.g., physical, chemical hazards, or sedentarism), air pollution, maternal and offspring health [66], racism and discrimination [67], and childhood adversity and trauma [68]. Addressing the interaction from preconception to lifetime exposures with pre-disposed factors (such as genetics and race) may provide a more robust understanding of an individual’s health outcomes [69, 70]. Every person has a unique totality of exposures, which affects how or why they develop a disease. According to Hill-Briggs et al. the social determinants of health are classified as socioeconomic status, neighborhood and physical environment, food environment, healthcare, and social context, each classification having three subfactors [71]. These social determinants of health are based on the circumstances of an individual’s life; they are “the conditions in which people are born, grow, work, live, and age, and the wider set of forces and systems shaping the conditions of daily life” [72], and therefore contribute in shaping the people’s exposures and health status. While there are common factors that help prevent disease at a population level, holistic and epidemiological evaluation of the exposome at the individual level may help uncover novel factors that could explain the CVH paradox.

Furthermore, the exposome differs significantly between racial groups, yet, the risk factors associated with CVD are failing to consider ethnic or geographical differences [73]. Although establishing basic guidelines for CVD prevention is crucial, the health disparities created by socio-demographics and economic status can influence the development of CVD. For example, the AHA CVH risk score does not distinguish between high-middle income countries (HMICs) and low-middle-income countries (LMICs). Most guidelines are based on the average US or European populations without the consideration of variability in neighborhood socioeconomic circumstances, or the contribution of LMICs. According to Yeates et al., applying an individualized-risk prevention program in LMICs has proven to be more cost-effective in reducing the risk of developing chronic diseases among high-risk individuals; however, such methods are unable to reach a wide-range of people like the generalized population-wide method does [74]. Using a combined approach for CVD risk reduction, i.e., both personalized and community-based prevention in a life course approach, both high- and low- risk individuals could be targeted in different geographical settings, all the while considering each individual’s social, economical, psychosocial, and ethnic backgrounds [75].

## Discussion

The cardiovascular health (CVH) paradox, where individuals with optimal Life’s Simple 7 (LS7) scores still develop cardiovascular disease (CVD) while others with poor CVH do not, highlights the limitations of current risk assessment models. Our review underscores the importance of addressing misclassification bias, cumulative exposure to risk factors, and incorporating novel elements in CVH assessments to better predict and prevent CVD.

### Misclassification Bias

Misclassification bias in self-reported lifestyle factors is a significant challenge in CVH assessment. Individuals often over-report healthy behaviors, leading to inflated LS7 scores and misrepresentation of true cardiovascular risk. Objective measures, such as biochemical verification of smoking status and wearable devices for physical activity tracking, could reduce this bias. Implementing these measures in routine clinical practice and large epidemiological studies would enhance the accuracy of CVH assessments and help mitigate the paradox.

### Cumulative Exposure to Risk Factors

Our findings suggest that cumulative exposure to risk factors over time is a more accurate predictor of CVD than single-point assessments. For instance, long-term exposure to elevated LDL cholesterol and blood pressure has a stronger association with cardiovascular events. This insight emphasizes the need for longitudinal monitoring and a life course approach to CVH assessment. Healthcare providers should consider cumulative risk in their prevention strategies, focusing on sustained lifestyle interventions and regular monitoring of risk factors.

### Personalized Prevention Elements

Incorporating personalized prevention elements offers promising avenues to address the CVH paradox:

1. **Markers of Subclinical Vascular Disease:** Early detection of subclinical vascular changes, such as arterial stiffness and endothelial dysfunction, can identify individuals at high risk for CVD before clinical symptoms appear. Routine screening using these markers could improve early intervention strategies and prevent disease progression.
2. **Blood and Urine Metabolic Biomarkers:** The inclusion of inflammatory and metabolic biomarkers in CVH assessments can provide additional predictive value for CVD. These biomarkers can help identify high-risk individuals who may benefit from more aggressive preventive measures.
3. **Emerging Risk Factors:** Social determinants of health, environmental exposures, and psychological stress significantly impact CVH. Addressing these factors through public health policies and individualized care plans can enhance cardiovascular outcomes. For example, reducing air pollution and improving access to healthy foods can have a substantial impact on population health.
4. **Polygenic Risk Scores (PRS):** Genetic predisposition plays a crucial role in CVD risk. PRS can identify individuals with a high genetic risk for CVD, allowing for early and targeted interventions. Combining PRS with traditional risk factors can refine risk stratification and personalize prevention strategies.
5. **Epigenetics:** Epigenetic modifications provide a link between environmental exposures, lifestyle factors, and CVD risk. Understanding these mechanisms can lead to new therapeutic targets and personalized prevention approaches. For instance, interventions targeting specific epigenetic changes could potentially reverse adverse effects and reduce CVD risk.
6. **Exposome:** A comprehensive evaluation of the exposome, including all environmental exposures over a lifetime, offers a holistic view of CVD risk. Integrating exposome data with genetic and biological factors can improve the understanding of individual susceptibility to CVD and guide personalized prevention strategies.

### Implications for Clinical Practice and Public Health

The integration of personalized prevention elements into CVH assessments has significant implications for clinical practice and public health. Personalized approaches can lead to more accurate risk predictions, enabling tailored interventions that address individual risk profiles. This shift towards precision medicine in CVD prevention can improve patient outcomes and reduce healthcare costs by preventing disease progression and complications.

Future research should focus on developing and validating comprehensive risk models that incorporate traditional risk factors, subclinical markers, metabolic biomarkers, genetic information, and environmental exposures. Large-scale studies and clinical trials are needed to establish the effectiveness of these personalized prevention strategies in diverse populations.

### Conclusion

Future studies are needed to gain more insight into the CVH paradox by: 1) describing the prevalence of the CVH paradox; and 2) investigating the mechanisms underlying the CVH paradox by evaluating a panel of potential causes across various geographical settings.

The achievement of these goals would require optimal identification of high CVD risk patients, at early stages of life and as precisely as possible (notion of “full health maintenance from before birth to golden years”). Therefore, it is crucial to combine the traditional risk factors with subclinical and circulating intermediary biomarkers, genetics, epigenetics and more broadly the exposome of each individual.

The growing accessibility of detailed information such as polygenic risk scores holds the promise to shift the perspective of CVD prevention towards a more individualized practice, although more progress needs to be made to render affordable and accessible mass assessment of other emerging markers. It is of primary importance for public health that the CVH paradox be better understood and addressed in order to reduce CVD morbidity and mortality. Prevention specialists have recognized the importance of applying a more individualized preventive care, yet, it remains to be acted upon and given more awareness for concrete implementation [75]. In order for personalized prevention to succeed, it is necessary to understand the mechanisms behind the approach and to ensure effective communication is being upheld between researchers, physicians, and patients. Widespread adoption of personalized prevention is a major untaking that requires both an international and a multidisciplinary effort. Specifically, personalized prevention is a public health priority, that would require involvement and support from politicians, public health officials, non-governmental organizations, scientists, epidemiologists, clinicians, and community members and stakeholders. Although it may be difficult and costly to carry out such a precise method of prevention in the short-term, implementation strategies should be uncomplicated, inexpensive with a long-term perspective to maximize sustainability impact that includes lowering the overall healthcare and economic burden that is associated with CVD.
